# Supplementary material for: Elimination of Eight Viruses and Two Viroids from Preclonal Candidates of Six Grapevine Varieties (Vitis vinifera L.) through In Vivo Thermotherapy and In Vitro Meristem Tip Micrografting
Source: Plants (Basel). 2022 Apr 13;11(8):1064. doi: 10.3390/plants11081064 (PMC9029751; doi:10.3390/plants11081064)
Supplement: Supplementary file 1 [file plants-11-01064-s001.zip › Supplementary Material/Supplementay Figure 1.pdf]

plants

Supplementary Material

Elimination of eight viruses and two viroids from preclonal candidates of six grapevine varieties (*Vitis vinifera* L.) through *in vivo* thermotherapy and *in vitro* meristem tip micrografting

Vanja Miljanić [1], Denis Rusjan [1], Andreja Škvarč [2], Philippe Chatelet [3] and Nataša Štajner [1,*]

[1]Department of Agronomy, Biotechnical Faculty, University of Ljubljana, 1000 Ljubljana, Slovenia; vanja.miljanic84@gmail.com; denis.rusjan@bf.uni-lj.si
[2]Chamber of Agriculture and Forestry of Slovenia, Agriculture and Forestry Institute Nova Gorica, 5000 Nova Gorica, Slovenia;
andreja.skvarc@go.kgzs.si
[3]UMR AGAP Institut, Univ Montpellier, CIRAD, INRAE, Institut Agro, F-34398 Montpellier, France; philippe.chatelet@inrae.fr
*Correspondence: natasa.stajner@bf.uni-lj.si; Tel.: +386 1 3203255

(**a**) GRSPaV

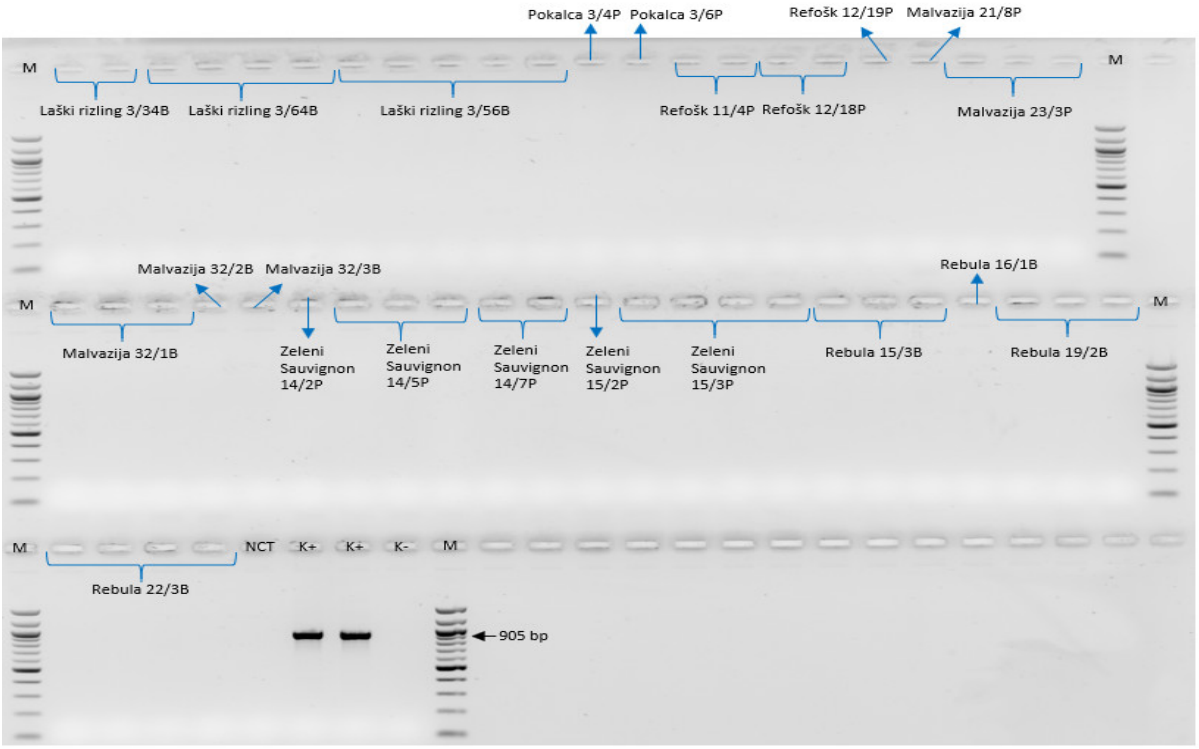

(b) GPGV

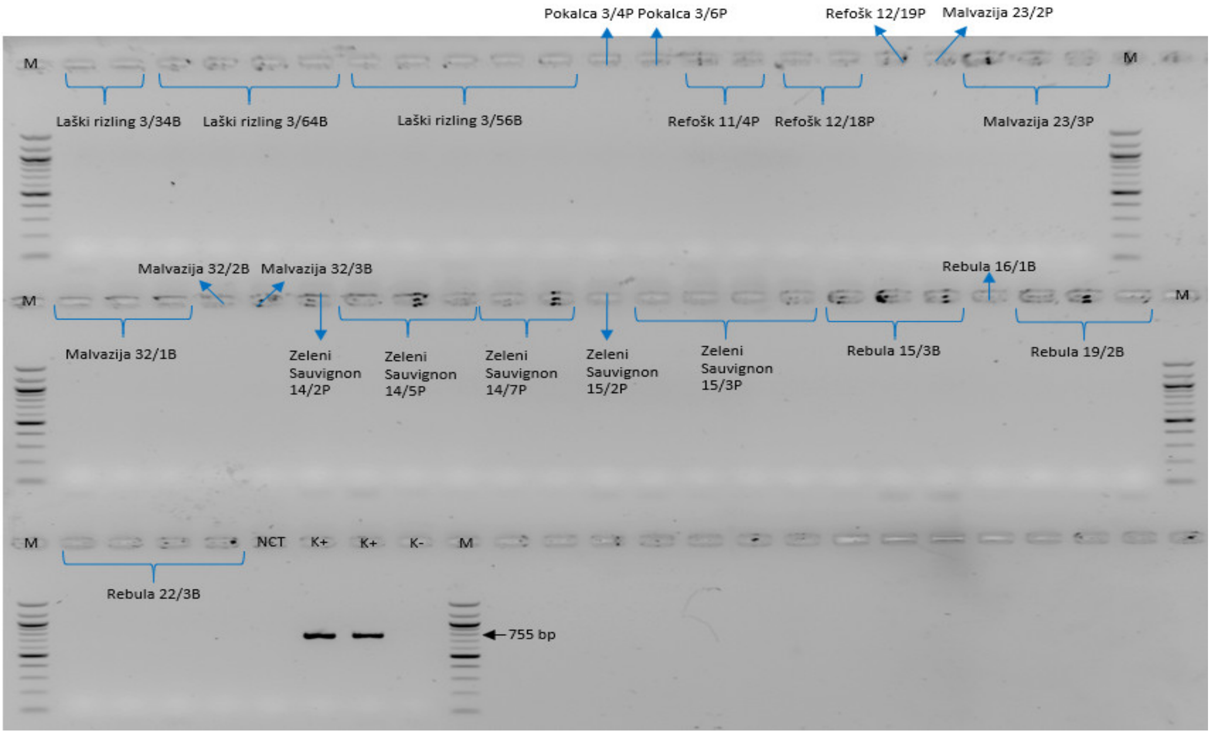

(**c**) GFLV

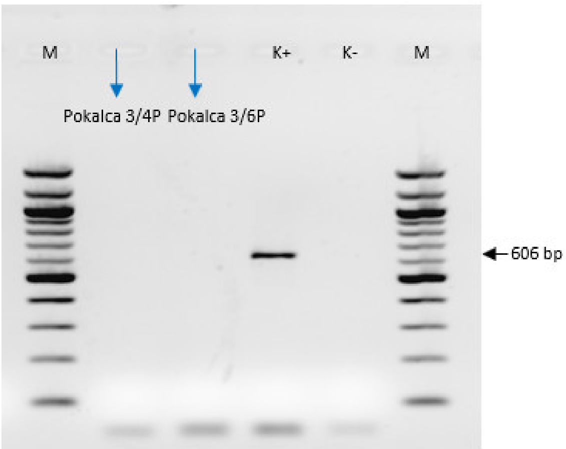

(**d**) GLRaV-3

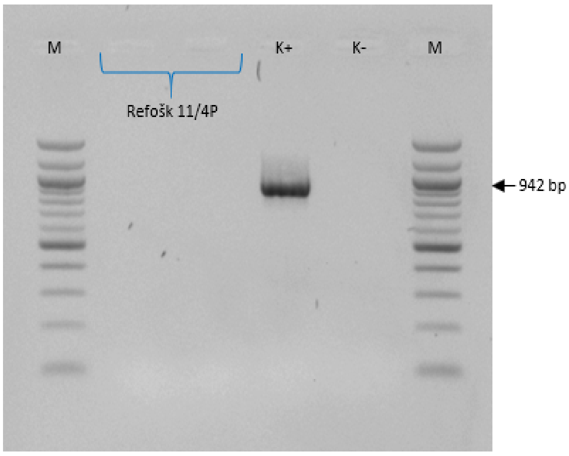

(**e**) GFkV

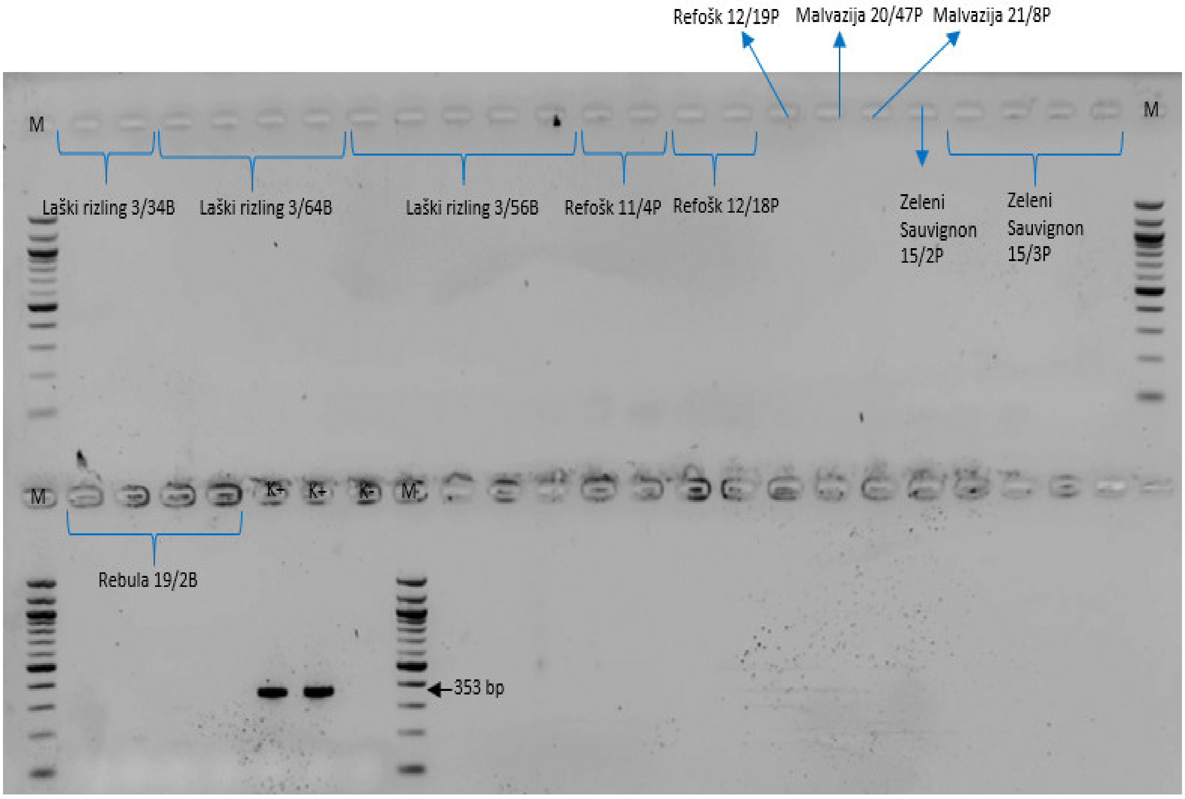
Refošk 12/19P
Malvazija 20/47P
Malvazija 21/8P
M
M
Laški rizling 3/34B
Laški rizling 3/64B
Laški rizling 3/56B
Refošk 11/4P
Refošk 12/18P
Zeleni Sauvignon 15/2P
Zeleni Sauvignon 15/3P
M
K+
K+
K-
M
Rebula 19/2B
353 bp

(**f**) GRVFV

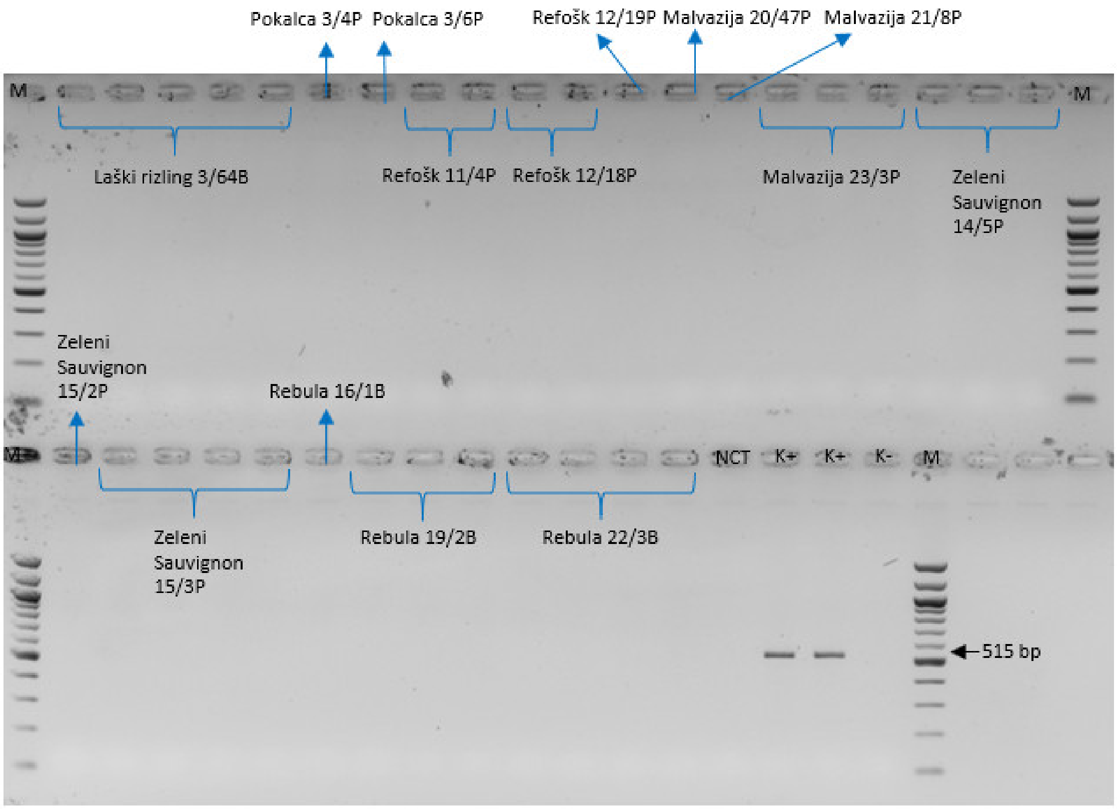

(**g**) GSyV-1

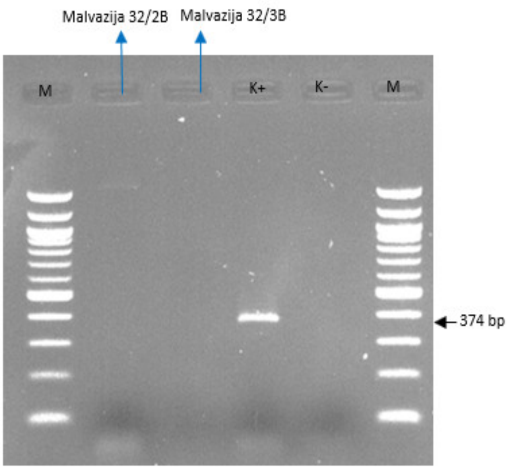

(**h**) RBDV

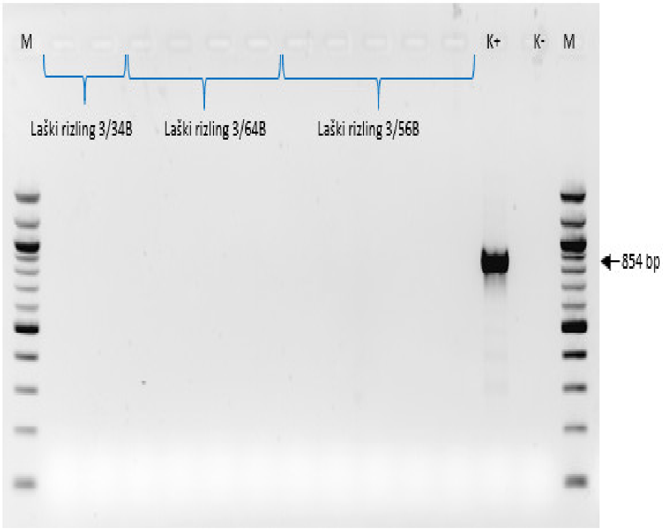

(**i**) HSVd

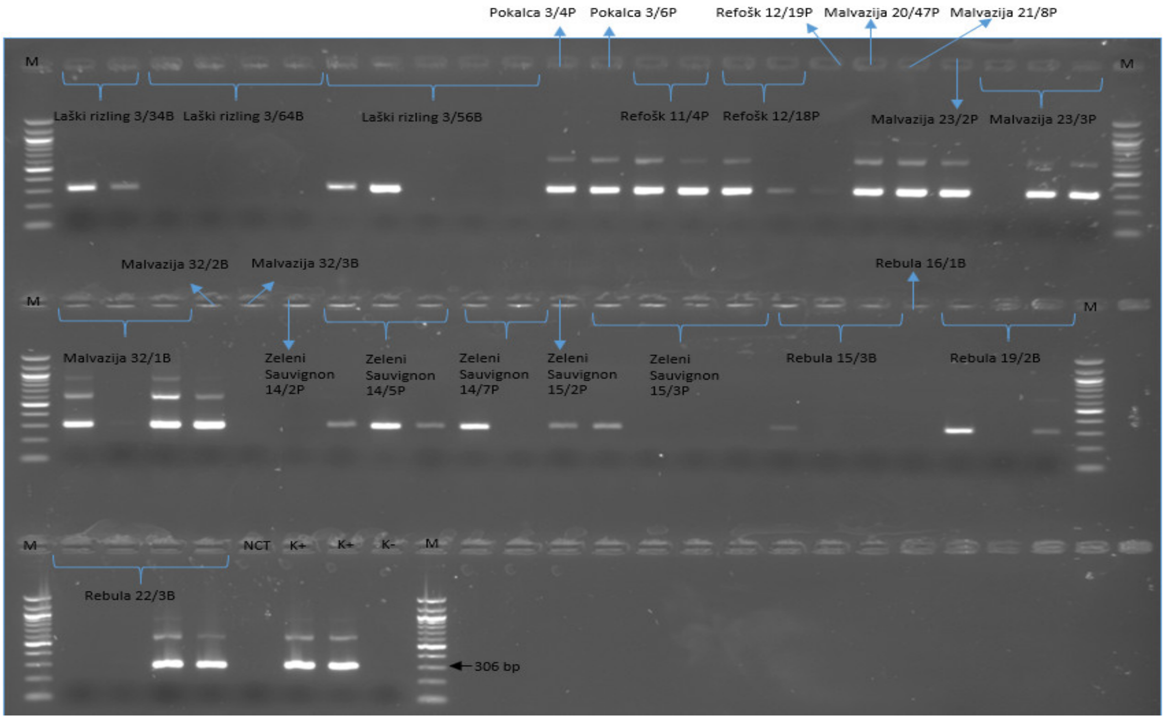

(**j**) GYSVd-1

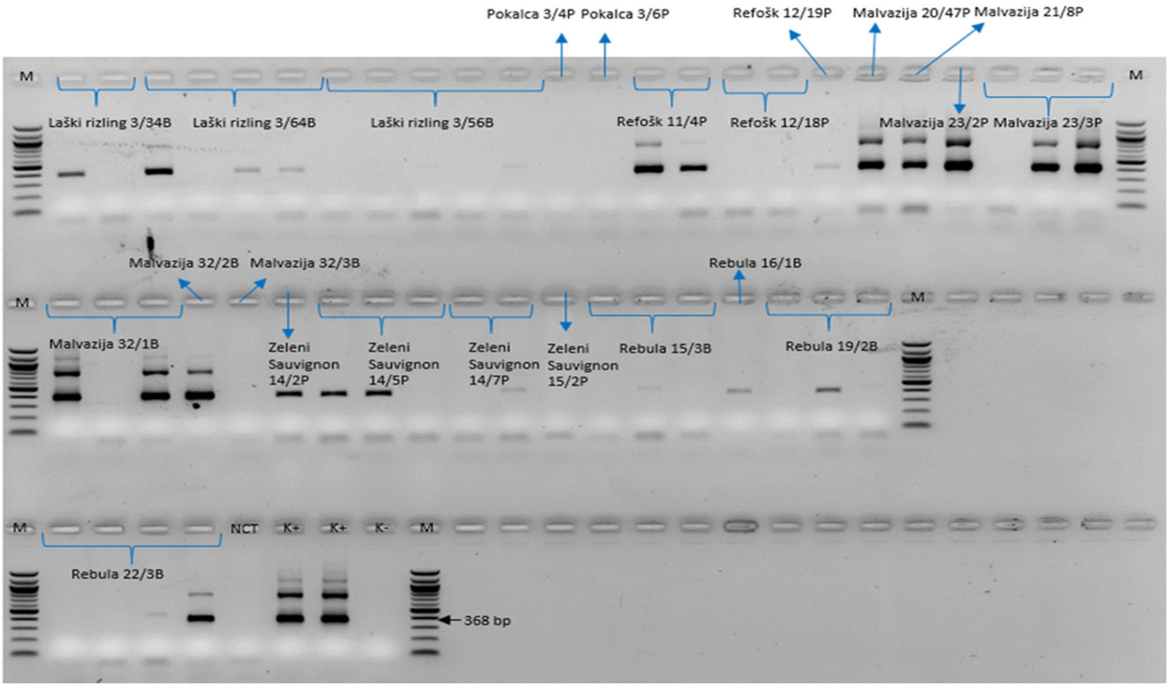

Supplementary Figure S1. Agarose gels of eight viruses and two viroids after sanitation process: (**a**) GRSPaV; (**b**) GPGV; (**c**) GFLV; (**d**) GLRaV-3; (**e**) GFkV; (**f**) GRVFV; (**g**) GSyV-1; (**h**) RBDV; (**i**) HSVd (in addition to well visible bends, very weak bends were observed for one sample of preclonal candidate Refošk 12/18P and one sample of preclonal candidate Malvazija 32/1B); (**j**) GYSVd-1 (in addition to well visible bends, very weak bends were observed for two samples of preclonal candidate Laški rizling 3/56B, one sample of preclonal candidate Rebula 15/3B and one sample of preclonal candidate Rebula 19/2B). M- Quick-Load Purple 100 bp DNA Ladder; NCT-non-control template for reverse transcription reaction; K+/K- positive/negative controls.
